# A Brief Tool to Assess Image-Based Dietary Records and Guide Nutrition Counselling Among Pregnant Women: An Evaluation

**DOI:** 10.2196/mhealth.6469

**Published:** 2016-11-04

**Authors:** Amy M Ashman, Clare E Collins, Leanne J Brown, Kym M Rae, Megan E Rollo

**Affiliations:** ^1^ School of Health Sciences Faculty of Health and Medicine University of Newcastle Callaghan Australia; ^2^ Priority Research Centre in Physical Activity and Nutrition Faculty of Health and Medicine University of Newcastle Callaghan Australia; ^3^ Gomeroi gaaynggal Centre Faculty of Health and Medicine University of Newcastle Tamworth Australia; ^4^ Department of Rural Health Faculty of Health and Medicine University of Newcastle Tamworth Australia; ^5^ Priority Research Centre in Reproduction Faculty of Health and Medicine University of Newcastle Callaghan Australia; ^6^ Mothers and Babies Research Centre Faculty of Health and Medicine University of Newcastle New Lambton Heights Australia

**Keywords:** nutrition assessment, pregnancy, telehealth, image-based dietary records

## Abstract

**Background:**

Dietitians ideally should provide personally tailored nutrition advice to pregnant women. Provision is hampered by a lack of appropriate tools for nutrition assessment and counselling in practice settings. Smartphone technology, through the use of image-based dietary records, can address limitations of traditional methods of recording dietary intake. Feedback on these records can then be provided by the dietitian via smartphone. Efficacy and validity of these methods requires examination.

**Objective:**

The aims of the Australian Diet Bytes and Baby Bumps study, which used image-based dietary records and a purpose-built brief Selected Nutrient and Diet Quality (SNaQ) tool to provide tailored nutrition advice to pregnant women, were to assess relative validity of the SNaQ tool for analyzing dietary intake compared with nutrient analysis software, to describe the nutritional intake adequacy of pregnant participants, and to assess acceptability of dietary feedback via smartphone.

**Methods:**

Eligible women used a smartphone app to record everything they consumed over 3 nonconsecutive days. Records consisted of an image of the food or drink item placed next to a fiducial marker, with a voice or text description, or both, providing additional detail. We used the SNaQ tool to analyze participants’ intake of daily food group servings and selected key micronutrients for pregnancy relative to Australian guideline recommendations. A visual reference guide consisting of images of foods and drinks in standard serving sizes assisted the dietitian with quantification. Feedback on participants’ diets was provided via 2 methods: (1) a short video summary sent to participants’ smartphones, and (2) a follow-up telephone consultation with a dietitian. Agreement between dietary intake assessment using the SNaQ tool and nutrient analysis software was evaluated using Spearman rank correlation and Cohen kappa.

**Results:**

We enrolled 27 women (median age 28.8 years, 8 Indigenous Australians, 15 primiparas), of whom 25 completed the image-based dietary record. Median intakes of grains, vegetables, fruit, meat, and dairy were below recommendations. Median (interquartile range) intake of energy-dense, nutrient-poor foods was 3.5 (2.4-3.9) servings/day and exceeded recommendations (0-2.5 servings/day). Positive correlations between the SNaQ tool and nutrient analysis software were observed for energy (ρ=.898, *P*<.001) and all selected micronutrients (iron, calcium, zinc, folate, and iodine, ρ range .510-.955, all *P*<.05), both with and without vitamin and mineral supplements included in the analysis. Cohen kappa showed moderate to substantial agreement for selected micronutrients when supplements were included (kappa range .488-.803, all *P* ≤.001) and for calcium, iodine, and zinc when excluded (kappa range .554-.632, all *P*<.001). A total of 17 women reported changing their diet as a result of the personalized nutrition advice.

**Conclusions:**

The SNaQ tool demonstrated acceptable validity for assessing adequacy of key pregnancy nutrient intakes and preliminary evidence of utility to support dietitians in providing women with personalized advice to optimize nutrition during pregnancy.

## Introduction

Dietitians can assess individual dietary needs and provide advice to clients to optimize their nutritional status [1]. In order to deliver personalized nutrition interventions, accurate information about what individuals are eating is required. For collection of such information to be feasible, the dietary data need to be collected and interpreted with a minimum burden on both the client and dietitian [2]. Feedback should be tailored to the individual and provided in a manner that is meaningful to the recipient so as to encourage positive dietary changes.

Traditional prospective methods of dietary assessment, including weighed or estimated food records, require the recording of all food and drinks consumed. These methods can capture day-to-day variation in diets and are used commonly in research [3]. However, keeping food records is associated with a high participant burden involved in the weighing or estimating of foods, may trigger changes in usual eating behaviors [4,5], and requires high levels of motivation to complete records accurately [3]. Reliability of written records decreases over time due to respondent fatigue, especially for recording periods of more than 4 days [6]. Keeping food records also requires literacy and numeracy skills and therefore may not be appropriate for all population groups. In clinical practice, retrospective methods of dietary assessment, such as diet histories and 24-hour food recalls, are more likely to be used. However, self-report places the onus on individuals to estimate food quantities consumed, a limitation that contributes to underreporting [7,8].

Manual analysis of food records by dietitians or other trained individuals is often required to translate reported food intakes into nutrients and food groups. This analysis is usually undertaken using food composition tables, often embedded in food analysis software. Food composition tables provide detailed information on nutrient composition of foods and drinks, giving determined values for quantities of energy, macronutrients (carbohydrate, protein, and fat), micronutrients (vitamins and minerals), and other food components, such as fiber [9].

Once dietary intake is analyzed, nutrient intakes can be compared with national recommendations. In Australia, the *Nutrient Reference Values* (NRVs) provide national intake recommendations for macro- and micronutrients [10]. The *Australian Guide to Healthy Eating* (AGTHE) is a visual food selection guide providing a representation of the proportions of food groups recommended for daily consumption [11]. The AGTHE supports the *Australian Dietary Guidelines* recommendation to “enjoy a wide variety of nutritious foods from these five food groups every day:” grain and cereal foods, vegetables, fruit, meat or alternatives (“meat”), and dairy or alternatives (“dairy”) [11]. The AGTHE is used as an educational and counselling tool by Australian dietitians to advise on the recommended number of daily servings from each food group and serving sizes from core nutrient-dense and noncore or discretionary energy-dense, nutrient-poor foods.

Innovative dietary assessment methods can address some of the limitations associated with current methods in order to improve the quality of data collected and ease of analysis. Image-based dietary records are a novel method for food and nutrient intake assessment [2,3], where images of consumed food and drinks capture a dietary record from which a person’s intake is determined [12]. A passive or active approach can be taken to capturing food intake. A passive approach involves wearable cameras that capture eating and drinking occasions [13,14]. While no effort from users is needed, privacy issues associated with this technology make passive methods of image capture challenging to implement. Active methods involve recording dietary intake via stand-alone cameras or those imbedded in handheld devices, such as smartphones. Although the active method relies on participants to capture the images, the burden of estimating portion size is placed on the dietitian or skilled person performing the analysis [15]. Smartphone ownership is increasing, with 77% of Australian adults owning a smartphone in 2015 [16]. Smartphone features such as cameras, microphones, and Internet connectivity make them an ideal mode of dietary assessment, education, and counselling. With access to appropriate technologies and training, dietary intake data can be relayed between clients and dietitians in real time, transcending distance, and potentially overcoming barriers relating to literacy or numeracy skills. These assessment methods support the provision of dietary feedback over distance (eg, through telephone or video consultation), broadening the scope of dietetic services [17]. Practical tools can support the use of image-based dietary records for both the collection of information on dietary intake and the analysis and interpretation of food and nutrient intake data. However, their use in clinical settings is limited if these tools are not convenient, and validation is required to support manual analysis of image-based dietary records by dietitians.

Previous methods of image-based dietary assessment have been examined in healthy adult [18-22], adolescent [23,24], and child [25] populations, in overweight and obese adults [26], and in type 2 diabetes [15,27]. To our knowledge, no studies to date have examined the use of image-based dietary records in pregnant women or in Indigenous Australians. Dietary intake and nutritional status during pregnancy have important implications for fetal development and growth, and the long-term health of both mother and infant [28-30]. In Australia, women of childbearing age are at risk of not meeting targets for recommended dietary intake (RDI) [31,32]. In particular, Indigenous women may experience structural barriers to optimal nutrition, including economic and geographical constraints to accessing food, and gaps in knowledge for choosing and preparing nutritious foods [33]. Novel lower-burden methods for dietary assessment and provision of feedback on nutrition warrant investigation and may be of benefit in these population groups.

The Diet Bytes and Baby Bumps (DBBB) study used image-based dietary records, captured via smartphone, in pregnant Indigenous and non-Indigenous women. The DBBB study sought to assess intake of AGTHE core and energy-dense, nutrient-poor food groups, total energy, and selected micronutrients, and to provide personalized feedback to these women via their smartphones, in combination with consultation with a dietitian.

The aims of this analysis were to evaluate the use of a brief approach to dietary analysis using a purpose-built Selected Nutrient and Diet Quality (SNaQ) tool to (1) assess nutrient intakes of pregnant women in the DBBB study, (2) assess the validity of the SNaQ tool for nutrient assessment relative to analysis using nutrient analysis software, and (3) assess the acceptability of SNaQ to pregnant women for provision of feedback on dietary intake.

The DBBB study was approved by the Aboriginal Health and Medical Research Council Ethics committee (962/13), Hunter New England Human Research Ethics Committee (13/06/19/4.04), and the University of Newcastle Human Research Ethics Committee (H-2013-0185). The study was conducted in two locations in New South Wales (NSW), Australia: Newcastle, the second largest city in NSW, and Tamworth, a regional inland NSW town.

## Methods

### Participants and Recruitment

We recruited participants via promotional fliers at hospital antenatal and general practitioner clinics and the University of Newcastle, through social media (including parenting sites), and through direct contact with pregnant women at antenatal clinics. In Tamworth, participants were also invited to participate through the Gomeroi gaaynggal Centre [34], an Indigenous research and ArtsHealth center. Participants were eligible if they were ≥18 years old, ≤24 weeks’ gestation, lived in Newcastle or Tamworth, had no current medical conditions, owned a smartphone, and were willing to use it to record their dietary intake for 3 days.

### Surveys and Study Timeline

**Figure 1:**
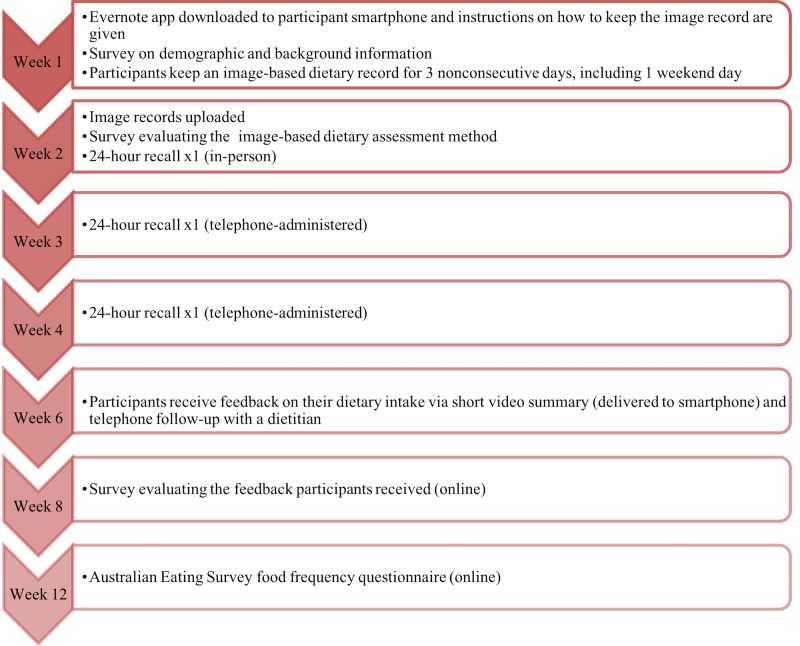
Diet Bytes and Baby Bumps study protocol.

The study ran for 12 weeks (Figure 1). Participants collected image-based dietary records in week 1, completed three 24-hour food recalls (in weeks 2, 3, and 4), received feedback on their dietary intake in week 6, and completed the Australian Eating Survey food frequency questionnaire in week 12 [35]. Participants completed 3 online surveys over the course of the study to provide demographic and background data (week 1, in-person study visit), evaluate the image-based dietary assessment method (week 2, in-person study visit), and evaluate the feedback on dietary intake that participants received (week 8, survey link sent via email).

### Diet Bytes Method

**Figure 2:**
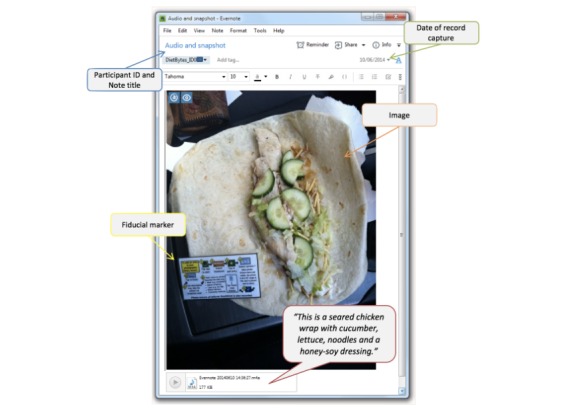
Example of an image-based dietary record in the Diet Bytes and Baby Bumps study, consisting of image, fiducial marker, and audio description of the food and drink items.

We modelled the method of capturing dietary intake using image-based records on our previous validated method in adults with type 2 diabetes [15,27]. However, in this study, to record dietary intake, participants used Evernote (Evernote Corporation, Redwood City, CA, USA), a free file-sharing and note-taking app for computers and smartphones. The Evernote app was downloaded onto participants’ smartphones during the first appointment. Participants were not expected to have any prior experience using the Evernote app. They were provided with training at the first appointment on how to use the app to record dietary intake and completed a test entry. The app was used to capture each eating occasion through notes or entries into a notebook (the dietary record). For the purpose of this study, the study team set up a shared notebook to allow the entries to be recorded. This notebook could only be viewed by the individual study participant and the research team who had access to the Evernote Diet Bytes account. We adjusted settings for Evernote so that the contents of the notebook were shared only with the research team over a Wi-Fi connection, so as not to use participants’ data. Participants also had the option of disabling their home Wi-Fi connection during the collection period (week 1), with the images then transmitted during the second study appointment (week 2) over the research center’s Wi-Fi connection. Participants were asked to collect information on all food, drinks, and nutritional supplements, such as prenatal vitamin and minerals, consumed over 3 nonconsecutive days, including 1 weekend day. Each eating occasion consisted of a note taken through the app, including an image of the food or drink items for consumption, with a fiducial marker (reference object) placed next to the items. Participants were also required to annotate a text or voice description, or both, of the image’s contents with information relating to cooking methods, brands, and types of foods (Figure 2). Any food or drink not consumed was captured using the same process. Participants were encouraged to label each eating occasion at data entry (eg, “Breakfast day 1”). However, the Evernote app automatically captured the date and time when records are made, which assisted with determining when meals were consumed.

### The SNaQ Tool

The SNaQ tool was developed as a brief tool to analyze participants’ dietary intake relative to AGTHE daily servings of core and energy-dense, nutrient-poor foods. We estimated key nutrients important during pregnancy (folate, calcium, iron, zinc, and iodine) based on average nutrient composition of the food group servings, using the Australian Food, Supplement & Nutrient Database (AUSNUT) 2007 [36] food composition tables embedded in the SNaQ tool, plus nutrients from micronutrient supplements consumed.

**Figure 3:**
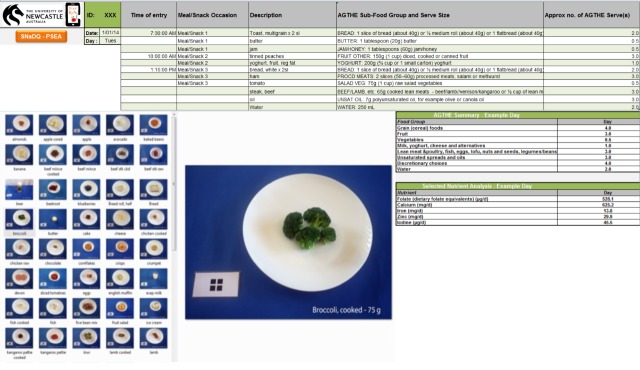
The Selected Nutrient and Diet Quality (SNaQ) analysis tool and portion size estimation aid (PSEA) for analysis of image-based dietary records in the Diet Bytes and Baby Bumps study. AGTHE: *Australian Guide to Healthy Eating*.

A portion size estimation aid (PSEA) included in the tool assisted with portion size quantification. The PSEA contained 80 photographs of a variety of AGTHE foods and drinks displayed in recommended serving sizes. The dietitian analyzing food portions compared the image from the image-based dietary record with images in the PSEA, in order to quantify portion size of the food and drink items in terms of number of AGTHE servings (see Figure 3). The text or voice description supplementing the image-based record further assisted with quantification. Mixed dishes and meals were broken down into their composite food groups. The image-based dietary records were first analyzed separately by 2 dietitians, who later conferred to confirm participant dietary intakes.

Feedback was provided to participants in week 6 of the study, via a short (1 minute) video designed to relay a simple, visual summary of food group intake compared with AGTHE recommendations. The video was transmitted to the Diet Bytes notebook, through the Evernote app on participants’ smartphones. Participants were sent a text message informing them that their feedback was available to view. The video could be paused and replayed as often as desired. Participants were given a few days to view their feedback and were then contacted later in the week by a dietitian for a telephone consultation. In the telephone conversation, results were discussed in greater detail, including core and energy-dense, nutrient-poor food group results and intakes of selected nutrients, to provide practical tailored examples of foods and serving sizes to optimize the participant’s pregnancy dietary intake.

### Statistical Analysis

We entered image-based dietary records into the nutrient composition software FoodWorks Professional version 7.0.3016 (Xyris Software [Australia] Pty Ltd) using the nutrient composition tables AUSNUT 2007 [36] (with “foods,” “brands,” and “supplements” selected). The PSEA assisted with the portion size estimation of the images for the FoodWorks entry using the same approach as for the SNaQ analysis, including the use of the image and text description for clarification of quantities, types, and brands of food and cooking methods. Data entered into the SNaQ tool, including information on the estimation of portion size, were not used during the analysis of the image-based records in FoodWorks software. We developed a protocol to standardize the entry of image-based dietary records into FoodWorks, including common assumptions made. For example, if the amount of butter or margarine on a piece of bread was unspecified, we assumed 1 teaspoon per slice and used the “not further specified” option for food types where possible when further details were not provided. Intraclass correlation coefficients for FoodWorks entries of the image-based records between 2 dietitians for a subsample of 10 participant records showed substantial agreement for energy and the selected micronutrients iron, folate, calcium, iodine, and zinc, in the range of .79-.99, all *P*<.05. One dietitian subsequently entered all image records into FoodWorks. We ascertained relative validity of the SNaQ tool in estimating participants’ total energy and selected nutrient intakes by comparison with the FoodWorks nutrient assessment of the image-based dietary records, and assessed by the strength of the relationship using Spearman rank correlation coefficients (ρ) and agreement between the methods using Cohen kappa. Analyses were performed using IBM SPSS statistical software version 23.0 (IBM Corporation). We took an inductive approach to analyze short qualitative responses on participants’ perceived acceptability of the feedback received [37].

## Results

### Characteristics of Participants

We enrolled 27 women in the DBBB study, with a median (interquartile range) age 28.8 (27.5-32.5) years, with 1 participant withdrawing due to time constraints. Of the remaining 26 participants, all were born in Australia, 8 (31%) identified as being of Indigenous descent, and all spoke only English at home. At study enrollment, 4 (15%) participants smoked tobacco products. At enrollment, participants ranged from 6 to 24 weeks’ gestation, with a mean (SD) of 18 (5) weeks. A total of 4 participants were in their first trimester of pregnancy, and 22 in their second trimester. For 15 (58%) participants it was the first pregnancy; 14 (54%) participants had an undergraduate or postgraduate university degree; and 2 developed health conditions (gestational diabetes and anemia) during the study.

Over half (n=17, 65%) had received nutrition advice from a health professional previously, although only 5 (19%) had received advice from a dietitian. Other sources of nutrition advice came from a general practitioner (n=10, 38%), midwife (n=5, 19%), obstetrician (n=1, 4%), or an antenatal clinic (n=1, 4%). Advice received focused on use of multivitamin supplements (n=12, 46%), managing morning sickness (n=7, 27%), healthy eating throughout pregnancy (n=7, 27%), weight gain during pregnancy (n=5, 19%), healthy eating during breastfeeding (n=5, 19%), or breastfeeding (n=4, 15%). Participants had also accessed pregnancy nutrition information from other sources, including friends (n=11, 42%), nongovernment websites (n=11, 42%), family (n=10, 38%), government websites (n=9, 35%), smartphone apps (n=7, 27%), and community groups, including mothers’ groups (n=2, 8%); 3 (12%) participants had not accessed any of these sources of information. A total of 11 (42%) participants felt they had received enough information about healthy eating for themselves and their baby at the time of enrollment, 13 (50%) were unsure, and 2 (8%) said they had not received enough information.

All participants used their smartphones for sending text messages (short message service, SMS) (n=26, 100%), and the majority for receiving SMS (n=25, 96%), searching or browsing the Internet (n=25, 96%), making voice calls (n=24, 92%), taking photos (n=24, 92%), sending or uploading photos (n=24, 92%), using apps (n=22, 85%), and taking notes (n=20, 77%). Over half (n=16, 62%) used their smartphones for taking videos and 12 (46%) to send or upload these videos. The majority of participants (n=18, 69%) had an Apple iPhone, and 8 (31%) had a Google Android phone. Only 4 (15%) had used their smartphones for making voice recordings.

### Food Group Intakes of Pregnant Women

Of the 26 participants, 24 (92%) recorded on all 3 days of the image-based dietary record, 1 participant recorded 2 days, and 1 recorded only 1 day. The participant recording on only 1 day was subsequently excluded from further analyses, and therefore further results are for the 25 participants with dietary records adequate for analysis. We used average food group and micronutrient intakes from participants’ multiple-day image records for this analyses.

Table 1 summarizes intakes of core and energy-dense, nutrient-poor foods. Median intakes of core food groups were close to recommendations for fruit and dairy, but did not meet recommendations for grains and cereals, vegetables, or meat, and exceeded recommendations for energy-dense, nutrient-poor foods. All Indigenous participants and approximately half (n=8, 47%) of non-Indigenous participants met recommendations for 0-2.5 daily servings of unsaturated spreads and oils.

### Relative Validity of the SNaQ Tool for Nutrient Assessment

Table 2 reports the correlations (Spearman correlation coefficients) and agreement (Cohen kappa) between nutrient values assessed from the SNaQ tool and from nutrient analysis software. Agreement was not substantial between the two methods for total energy (kappa=.031, *P*=.67). Correlation coefficients for nutrient intakes assessed by the two methods of analyzing the image-based dietary records ranged from ρ=.791 to ρ=.955 (all *P*<.001) for key micronutrients (iron, folate, calcium, zinc, and iodine) when supplements were included in the analysis (kappa range .488-.803, all *P≤*.001). With supplement use excluded, correlations ranged from ρ=.510 to ρ=.888 (all *P*<.05). Agreement between the two analysis methods, ascertained via Cohen kappa, was significant for calcium (kappa=.544, *P*<.001), iodine (kappa=.632, *P*<.001), and zinc (kappa=.572, *P*<.001). Agreement was poor for folate when supplement use was not included (kappa=-.068, *P*=.52). Both the SNaQ tool and FoodWorks analyses identified that no participant met the estimated average requirement (EAR) for iron of 22 mg when supplement use was not included.

**Table 1:** Intake of core foods as assessed by the Selected Nutrient and Diet Quality (SNaQ) brief analysis tool from the Diet Bytes and Baby Bumps image-based dietary records (n=25).

Food group		Food group intake in servings/day		AGTHE^b^ recommended intake during pregnancy in servings/day	Meeting recommended intake of servings	
		Mean (SD)	Median (IQR^a^)		No. of servings	n (%)
**All participants combined (n=25)**						
	Grains and cereals	4.8 (2.0)	4.7 (3.6-6.5)	8.5	≥8.5	1 (4)
	Vegetables	2.4 (1.4)	2.2 (1.2-3.5)	5	≥5	1 (4)
	Fruit	1.9 (1.6)	1.7 (0.9-2.5)	2	≥2	10 (40)
	Lean meat	2.0 (1.0)	1.9 (1.4-2.9)	3.5	≥3.5	2 (8)
	Dairy	2.1 (1.3)	1.8 (1.3-2.7)	2.5	≥2.5	10 (40)
	Unsaturated spreads and oils	1.9 (1.4)	2.0 (0.5-3.0)	0-2.5	0-2.5	16 (64)
	Energy-dense, nutrient-poor foods	3.7 (1.9)	3.5 (2.4-3.9)	0-2.5	0-2.5	7 (28)
**Indigenous participants (n=8)**						
	Grains and cereals	4.7 (2.3)	4.3 (3.4-6.1)	8.5	≥8.5	1 (13)
	Vegetables	2.0 (1.4)	1.6 (1.1-3.2)	5	≥5	0 (0)
	Fruit	1.4 (1.9)	0.9 (0.0-2.3)	2	≥2	2 (25)
	Lean meat	1.6 (0.9)	1.5 (0.8-2.0)	3.5	≥3.5	0 (0)
	Dairy	2.5 (1.9)	2.3 (1.0-3.4)	2.5	≥2.5	4 (50)
	Unsaturated spreads and oils	0.8 (0.8)	0.7 (0.8-1.7)	0-2.5	0-2.5	8 (100)
	Energy-dense, nutrient-poor foods	4.1 (2.9)	3.7 (1.6-7.1)	0-2.5	0-2.5	2 (25)
**Non-Indigenous participants (n=17)**						
	Grains and cereals	4.9 (1.9)	4.9 (3.6-6.9)	8.5	≥8.5	0 (0)
	Vegetables	2.6 (1.4)	2.4 (1.7-3.5)	5	≥5	1 (6)
	Fruit	2.2 (1.4)	1.8 (1.4-2.7)	2	≥2	8 (47)
	Lean meat	2.2 (1.0)	2.0 (1.7-3.1)	3.5	≥3.5	2 (12)
	Dairy	1.9 (0.9)	1.7 (1.3-2.7)	2.5	≥2.5	6 (36)
	Unsaturated spreads and oils	2.3 (1.4)	2.8 (1.0-3.3)	0-2.5	0-2.5	8 (47)
	Energy-dense, nutrient-poor foods	3.5 (1.3)	3.5 (2.4-3.9)	0-2.5	0-2.5	5 (29)

^a^IQR: interquartile range (25th-75th percentiles).

^b^AGTHE: *Australian Guide to Healthy Eating* [11]. Examples of serving sizes of foods: grains and cereals (standard serving 500 kJ), eg, 1 slice of bread, 0.5 cup cooked grain; vegetables (standard serving 75 g, 100-350 kJ), eg, 0.5 cup cooked vegetables, 1 cup raw vegetables, 0.5 medium potato; fruit (standard serving 150 g, 350 kJ), eg, 1 medium piece, 2 small pieces, 125 mL fruit juice (no added sugar, only occasionally); lean meats and alternatives (standard serving 500-600 kJ), eg, 65 g cooked lean red meats, 80 g cooked lean poultry, 100 g cooked fish, 2 large eggs, 1 cup cooked legumes or beans; dairy and alternatives (standard serving 500-600 kJ), eg, 1 cup milk, 2 slices (40 g) hard cheese, 0.75 cup yoghurt, 60 g sardines; unsaturated spreads and oils (standard serving 250 kJ), eg, 10 g unsaturated spread, 7 g unsaturated oil, 10 g nuts; energy-dense, nutrient-poor foods (standard serving 600 kJ), eg, 2 scoops ice cream, 50-60 g processed meats, 1 can soft drink, 12 hot chips, 200 mL wine.

**Table 2:** Correlation and agreement for energy and selected nutrient intake from mean 3-day image-based dietary records in the Diet Bytes and Baby Bumps study (n=25 participants) analyzed by the Selected Nutrient and Diet Quality (SNaQ) tool and FoodWorks (FW) nutrient analysis software.

Nutrient		Method	Input, median (IQR^a^)	ρ (*P* value)	n (%) <EAR^b^	n (%) ≥EAR to <RDI^c^	n (%) ≥RDI	Cohen kappa (*P* value)
**Intake from food and supplements**								
	Energy (kJ/day)	SNaQ	8418.33 (7755.83-10,004.17)	.898 (<.001)	N/A^d^			.031^e^ (.67)
		FW	7738.89 (6329.94-8995.05)					
	Iron (mg/day)	SNaQ	11.30 (8.93-15.08)	.812 (<.001)	21(84)	0 (0)	4 (16)	.533 (<.001)
		FW	13.54 (10.75-21.47)		19 (76)	3 (12)	3 (12)	
	Calcium (mg/day)	SNaQ	877.36 (653.74-1181.60)	.791 (<.001)	12 (48)	4 (16)	9 (36)	.488 (.001)
		FW	831.01 (672.39-1000.89)		13 (52)	6 (24)	6 (24)	
	Folate, total DFE^f^ (µg/day)	SNaQ	851.90 (225.15-1156.15)	.893 (<.001)	11 (44)	1 (4)	13 (52)	.559 (.001)
		FW	820.20 (393.53-1383.00)		8 (32)	2 (8)	15 (60)	
	Iodine (µg/day)	SNaQ	167.00 (93.52-311.28)	.955 (<.001)	11 (44)	4 (16)	10 (40)	.803 (<.001)
		FW	171.42 (92.58-300.20)		12 (48)	3 (12)	10 (40)	
	Zinc (mg/day)	SNaQ	13.09 (10.46-19.56)	.905 (<.001)	3 (12)	4 (16)	18 (72)	.741 (<.001)
		FW	14.66 (10.24-21.24)		3 (12)	5 (20)	17 (68)	
**Intake from food only, supplements excluded**								
	Energy (kJ/day)	SNaQ	8418.33 (7755.83-10,004.17)	.898 (.000)	N/A			.031^e^ (.67)
		FW	7738.89 (6329.94-8995.05)					
	Iron (mg/day)	SNaQ	9.50 (7.70-10.85)	.510 (.009)	25 (100)	0 (0)	0 (0)	Constants (no statistics computed)
		FW	11.78 (8.53, 13.73)		25 (100)	0 (0)	0 (0)	
	Calcium (mg/day)	SNaQ	809.90 (653.75-1181.70)	.888 (<.001)	14 (56)	2 (8)	9 (36)	.554 (<.001)
		FW	736.61 (663.19-927.37)		17 (68)	3 (12)	5 (20)	
	Folate, total DFE (µg/day)	SNaQ	319.00 (240.25-433.35)	.600 (.002)	21 (84)	4 (16)	0 (0)	-.068 (.52)
		FW	409.79 (259.74-642.22)		16 (64)	2 (8)	7 (28)	
	Iodine (µg/day)	SNaQ	99.00 (79.80-139.05)	.850 (<.001)	22 (88)	2 (8)	1 (4)	.632 (<.001)
		FW	104.25 (86.46-130.95)		22 (88)	2 (8)	1 (4)	
	Zinc (mg/day)	SNaQ	10.60 (8.40-13.10)	.745 (<.001)	7 (28)	6 (24)	12 (48)	.572 (<.001)
		FW	10.63 (8.89-13.47)		6 (24)	9 (36)	10 (40)	

^a^IQR: interquartile range (25th-75th percentiles).

^b^EAR: estimated average requirement. EAR is a nutrient level estimated to meet the requirements of 50% of the healthy individuals in a life stage or gender group, per day (EARs for nutrients as follows: iron 22 mg, calcium 840 mg, folate 520 µg, iodine 160 µg, zinc 9 mg) [10].

^c^RDI: recommended dietary intake. RDI is the average dietary intake level sufficient to meet nutrient requirements of 97% to 98% of healthy individuals in a life stage or gender group, per day (RDIs for nutrients as follows: iron 27 mg, calcium 1000 mg, folate 600 µg, iodine 220 µg, zinc 11 mg) [10].

^d^N/A: not applicable.

^e^Kappa for energy intake in categories of 1000 kJ.

^f^DFE: dietary folate equivalents.

**Table 3:** Participant’s perceived acceptability for receiving dietary counselling in the Diet Bytes and Baby Bumps study (n=22^a^) (survey questions with agree-disagree responses).

Questions	Strongly agree n (%)	Agree n (%)	Neutral n (%)	Disagree n (%)	Strongly disagree n (%)
I believe that the combination of the summary of my dietary intake that I received via my mobile/smartphone and the follow-up with the dietitian was helpful.	12 (55)	9 (41)	1 (5)	0 (0)	0 (0)
The summary of my dietary intake that I received via my mobile/smartphone was easy to understand on its own. I did not need to speak to a dietitian to clarify.	2 (9)	5 (23)	6 (27)	8 (36)	1 (5)
The summary of my dietary intake that I received via my mobile/smartphone was difficult to understand.	0 (0)	2 (9)	3 (14)	13 (59)	4 (18)
Neither the summary of the results from the analysis of my photographic dietary record that I received on my mobile/smartphone nor the advice that I received from the dietitian was helpful.	0 (0)	1 (5)	1 (5)	4 (18)	16 (73)

^a^n=22. Two participants did not receive the telephone counselling (1 gave birth before it could be given and 1 did not respond to contact) and 2 participants did not answer this survey.

**Table 4:** Participant’s perceived acceptability for receiving dietary counselling in the Diet Bytes and Baby Bumps study (n=22^a^) (survey questions with yes/no responses).

Questions	Yes, n (%)	No, n (%)
I have changed my diet as a result of the nutrition advice that I received as part of this study.	17 (77)	5 (23)
I have changed the kinds of foods I eat.	16 (73)	6 (27)
I have changed the amount of food I eat.	8 (36)	14 (64)
I have changed the cooking methods I use.	3 (14)	19 (86)
I have changed how I keep track of what I eat and drink.	5 (23)	17 (77)
I have made other changes.	1 (5)	21 (95)

^a^n=22. Two participants did not receive the telephone counselling (1 gave birth before it could be given and 1 did not respond to contact) and 2 participants did not answer this survey.

### Acceptability of Receiving Feedback on Dietary Intake

Table 3 and Table 4 summarize participants’ perceived acceptability of receiving nutrition feedback. Over three-quarters (n=17, 77%) of the 22 participants who responded to the final survey reported that they had made dietary changes as a result of the personalized nutrition feedback. Changes to the type of foods consumed fell into three categories: (1) food groups or individual foods, including eating more red meat, vegetables, fruit, and individual foods like Milo, yoghurt, cheese, and crackers); (2) nutrients, including consuming more foods higher in iron, calcium, and protein, or continuing or starting to take a prenatal vitamin or mineral supplement; and (3) changes to eating behaviors, including increasing snack occasions. Some participants reported eating greater quantities of foods from the core food groups, while others reported eating smaller amounts of some unspecified foods, and consuming less soft drink and “junk” foods. Some participants reported changes to cooking methods related to meat and vegetables, such as steaming vegetables, and using cooking spray rather than oil or butter to cook meat. When asked if participants had changed how they monitored their dietary intake, 1 participant reported sometimes using an app (not Evernote) to record her intake, although this was a behavior in place prior to the study.

Some participants thought the advice from a dietitian was useful and helped to clarify the feedback provided via the video summary; for example:

...the phone consult was very useful to me. Without this the written feedback would have been far less meaningful. I did like the visual graphs to help me understand the information.

27 years old, first baby

Additionally, another participant commented:

It was very detailed and thorough and easier to understand what should be done to improve my diet compared to the diet summary received on Evernote.

32 years old, first baby

Others reported not making changes as a result of the feedback, due to already meeting requirements or not being able to fit all the recommended servings into their daily intake.

Some participants felt that DBBB could be improved by keeping the image-based records for a longer duration and by taking notes, rather than images, for certain foods such as snacks and water. More SMS reminders were requested, as some participants reported forgetting to take images. One commented that having to take an image before eating when you were hungry was inconvenient:

It’s inconvenient to take pictures of food before eating when hungry (which is most of the time), however I think this is a useful way of assessing dietary intake.

27 years old, first baby

The majority of respondents (n=18, 82%), preferred receiving nutrition feedback via the combination of the video summary and follow-up telephone consultation with a dietitian. One indicated that she preferred the video feedback alone, and 3 preferred the consultation with the dietitian alone. Only 1 participant indicated an alternative method for receiving nutrition advice, via a printable email summary.

## Discussion

We observed strong positive correlations between the SNaQ tool and the nutrient analysis software for estimates of total energy intake and all selected micronutrients (iron, calcium, zinc, folate, and iodine), both with and without micronutrient supplements included in the analysis. However, SNaQ overestimated energy intake compared with the FoodWorks analysis (8418 kJ vs 7739 kJ) and underestimated intakes of some micronutrients (iron, iodine, and zinc when supplements were included in the analysis; iron, folate, and iodine when supplements were excluded). The relatively minor differences in intakes were not clinically important differences, as evidenced by the comparison of classifications of nutrient intake adequacy (EAR, RDI) using Cohen kappa (in Table 2). Agreement is considered moderate if .41 ≤kappa ≤.6 and substantial if .61≤ kappa ≤.8 [38]. Cohen kappa indicated moderate agreement (kappa range .488-.559, all *P* ≤.001) between the two methods for assessing adequacy of nutrient intakes of iron, calcium, and folate, and substantial agreement for iodine (kappa=.803, *P*<.001) and zinc (kappa=.741, *P*<.001), when supplements were included in the analysis. When supplements were excluded from the analysis, there was moderate agreement for calcium (kappa=.554, *P*<.001) and zinc (kappa=.572, *P*<.001), and substantial agreement for iodine (kappa=.632, *P*<.001). Future estimation of bias could be explored through criterion validity (ie, comparison with objective measures of dietary intake such as nutritional biomarkers). However, this was beyond the scope of our study, which aimed to assess the relative validity of the SNaQ tool compared with nutrient intakes assessed using dietary composition software.

Specifically designed as a brief tool, the SNaQ tool therefore did not include all foods within the food composition database, and as such may have underestimated some micronutrients. When we removed supplements from the analysis, the SNaQ tool did not show significant agreement with the nutrient software analysis for folate (kappa=-.068, *P*=.52). While the nutrient software analysis indicated that 9 participants had nutrient intakes meeting or greater than the EAR of 520 µg, SNaQ showed that only 4 participants had intakes that met the EAR. This may be related to the inclusion of Vegemite (a yeast-based spread) in the image-based records of 7 participants (28%) who ate this food on at least 1 record day. A serving (5 g) of Vegemite provides 100 µg folate (19% of the pregnancy EAR) [39], and so pregnant women may be able to meet their requirements without supplements on certain days, if they consume specific folate-rich foods. As the aim of the SNaQ tool was to also provide a food group analysis of participant diets, we did not include some foods that do not fall into food group categories in the SNaQ (eg, gravy, Vegemite, tomato sauce and some other condiments, salt, and fortified foods like Nestlé Milo). This has highlighted that future modifications to SNaQ may be required to better reflect foods commonly consumed by pregnant women.

The majority of pregnant women in the DBBB study did not meet the recommended AGTHE target for daily servings of grain and cereal foods, vegetables, fruit, meat, and dairy. The median daily servings of unsaturated spreads and oils met recommendations, while median intakes of energy-dense, nutrient-poor foods exceeded recommendations, with less than a third of participants consuming within the target 0-2.5 servings/day. When we evaluated food intakes excluding micronutrient supplements, both the SNaQ and nutrient composition software showed that median intakes of selected key micronutrients important in pregnancy were lower than the EAR for iron, calcium, folate, and iodine. When we included vitamin and mineral supplements use, the median intake of iron was still below the EAR.

Intakes of energy-dense, nutrient-poor foods were high, with the majority (n=18, 72%) exceeding the maximum target of 2.5 servings/day. In other cohorts of pregnant Australian women, it has been reported that meeting AGTHE and NRV targets is challenging [40]. Pregnant women have higher requirements for some nutrients, including folate, iron, zinc, and iodine [10]. Australiawide in 2011-2012, 11.7% of nonpregnant women aged 19-30 years did not meet the EAR for iodine, 10.9% for folate, 13.5% for zinc, 37.5% for iron, and 71.3% for calcium [32]. In addition, within the Australian Longitudinal Study on Women’s Health (ALSWH) cohort, suboptimal intakes of core foods and nutrients in pregnant women were common [40,41]. Only 1.5% of the 606 pregnant participants achieved the NRVs for key micronutrients, with no pregnant woman meeting AGTHE target intakes for all food groups and median intakes of energy-dense, nutrient-poor choices exceeding recommendations [40]. However, ALSWH highlighted that women who consumed more daily servings of fruit and dairy than the AGTHE targets met pregnancy NRVs, as did consuming more than the 2.5 daily servings of energy-dense, nutrient-poor foods, although this was associated with higher total energy and saturated fat intakes [40]. This indicates that provision of personalized nutrition advice to optimize diet quality and nutrient intake in pregnancy is warranted. While AGTHE serving sizes and recommended numbers of servings have been revised since this time, including an increase in recommendations from 1.5 to 3.5 servings of meat, and an additional half serving of dairy foods, dietary intakes are still of concern. In the DBBB cohort the median intake from AGTHE food groups did not meet the revised targets for nonpregnant women, with less than 10% meeting targets for daily servings of meat (n=2, 8%), vegetables (n=1, 4%), and grain foods (n=1, 4%), and less than half meeting targets for fruit (n=10, 40%) and dairy (n=10, 40%).

Prior to being in the study, less than half (n=12, 46%) of participants had received information on prenatal nutrient supplements (including folic acid and iron) during their pregnancy, although the results of this study imply that micronutrient supplementation use may help women meet pregnancy EARs, particularly for iron, folate, and iodine. Only approximately a quarter of participants (n=7, 27%) had received advice on healthy eating during pregnancy prior to the study. Nutrition knowledge among pregnant women in Australia is suboptimal, with one cross-sectional study of 400 pregnant women showing that over half (65%) of participants were not familiar with AGTHE recommendations [42,43]. However, high motivation among pregnant women to adopt healthy eating behaviors [43] and increased awareness of nutrition during pregnancy [44] imply that pregnancy may be an opportune time for health professionals to intervene to improve women’s nutrition-related knowledge. The results from the DBBB study further suggest that pregnant women could potentially benefit from receiving personalized nutrient intake assessment and provision of information. This was supported by the less than half (n=11, 42%) of participants who reported they felt they had received enough information about healthy eating for themselves and their baby at the time of enrollment, and a high proportion of participants who reported changes to their dietary intake in response to receiving tailored feedback.

The majority (n=17, 77%) of participants who completed the final survey reported that they had made changes to their dietary intake as a result of receiving the personalized feedback, which consisted of the video summary and the telephone consultation with the dietitian. The preferred method of receiving dietary advice was from the video summary and the dietitian consultation combined, with 95% (n=21) of participants agreeing that this combined way of receiving feedback was helpful. Previous research in the area of apps for dietary feedback during pregnancy supports our findings in this study. A recent evaluation of a Dutch online coaching program delivered by a mobile health platform (called Smarter Pregnancy) resulted in improvements in vegetable, fruit, and folic acid intake in pregnant women, although these were not statistically significant, and high compliance with positive feedback from participants was reported [45]. Likewise, results from the pilot study of the Eating4two app, to monitor gestational weight, was viewed favorably by participants as a method to assist in supporting healthy pregnancy dietary behaviors [46]. Dietary advice during pregnancy can come from multiple sources (as reported by DBBB participants), and can be confusing and contradictory [47], and it is therefore promising that this method was perceived as acceptable by participants. Furthermore, the feedback received indicates that the Diet Bytes method is promising and warrants future testing in randomized controlled studies to establish the efficacy of using a personalized smartphone method for improving pregnancy food and nutrient intakes [48].

### Limitations

Limitations of our study include the small sample size of 25 participants completing the study protocol. A review [12] of image-based dietary assessment methods found that validation studies using this method to date have been conducted in sample sizes ranging from 9 [49] to 75 [19] participants. Given the small sample size and the wide variety of foods available in the Australian food supply, more days of recorded dietary intake may have been required to optimize accuracy of estimated food intake. In relation to the quantification of food portions contained in the image-based records, we attempted to reduce the introduction of bias during the analysis. Coding and entry of the records using the SNaQ tool and FoodWorks were performed independently at separate time points, and information on the estimations of portion size made using the SNaQ tool was not available to the dietitian during the FoodWorks analysis. Despite this, it is possible that estimations of portion size made using the SNaQ tool may have influenced the FoodWorks analysis for the first dietitian. However, the subsample (10 participants) analysis of the image-based records by a second dietitian (using FoodWorks only) showed high agreement with the analysis of the first dietitian, suggesting that any impact may have been small. At the time of developing the SNaQ tool, AUSNUT 2007 was the most recent nutrient composition database available, and this was embedded in the SNaQ tool and also used for the FoodWorks analysis. This database does not contain food group equivalents for each of the food items, and we therefore could not establish intakes of AGTHE food groups from FoodWorks. It is therefore a limitation of this study that we were unable to compare estimates of food group intakes between the two methods.

Participants in the DBBB study may not be representative of all Australian women. Those without smartphones were excluded from participating, and therefore these results may not be representative of women who are economically vulnerable or who have other reasons for not owning a smartphone. We did not collect data on prepregnancy weight and weight gain during pregnancy, and therefore we do not know whether study participants were achieving recommendations for appropriate pregnancy weight gain. The median age of DBBB study participants (28.8 years) was slightly lower than the NSW state median age of women giving birth in 2014 (31.2 years) [50] and may be indicative of the number of rural and Indigenous women in this study, who tend to have their children at a younger age [51]. Over half the participants had completed a university degree, compared with 29% of all women Australiawide of working age (15-64 years) in 2015 [52]. However, this study purposively recruited Indigenous participants (n=8, 31%), to ensure their representation in the study was adequate for separate analyses. It should be noted that Australiawide it is estimated that 3% of the population is Aboriginal or Torres Strait Islander [53]. Given that one of the recruitment avenues was through an Indigenous birth cohort to specifically target this population group, the high representation of Indigenous participants in this study is to be expected and is desirable given that the use of image-based dietary records has not been previously evaluated in this population.

### Conclusions

To our knowledge, this study is the first to evaluate the use of image-based dietary records for dietary assessment in pregnant women, including Indigenous Australian women, and demonstrated that the SNaQ tool can adequately assess key nutrient intakes during pregnancy. With training and practice, the SNaQ tool has the potential to be both time and resource saving as a dietary assessment tool for dietitians, while reducing the burden of recording associated with traditional methods for participants. Importantly this study highlights that using an image-based dietary record in combination with individual phone consultation with a dietitian for the provision of dietary feedback during pregnancy is acceptable. The Diet Bytes method for nutrition assessment and provision of personally tailored feedback may be a useful method for dietitians to assist women in optimizing their food and nutrient intakes during pregnancy.

## Abbreviations

AGTHE: Australian Guide to Healthy Eating
ALSWH: Australian Longitudinal Study on Women’s Health
AUSNUT: Australian Food, Supplement & Nutrient Database
DBBB: Diet Bytes and Baby Bumps
EAR: estimated average requirement
NRV: Nutrient Reference Value
NSW: New South Wales
PSEA: portion size estimation aid
RDI: recommended dietary intake
SMS: short message service
SNaQ: Selected Nutrient and Diet Quality
